# Loss of ING4 enhances hematopoietic regeneration in multipotent progenitor cells

**DOI:** 10.1371/journal.pone.0316256

**Published:** 2025-02-14

**Authors:** Georgina A. Anderson, Marco Hernandez, Carlos Alfaro Quinde, Zanshé Thompson, Vera Binder-Blaser, Alison M. Taylor, Katie L. Kathrein

**Affiliations:** 1 Department of Biological Sciences, University of South Carolina, Columbia, South Carolina, United States of America; 2 Department of Biomedical Engineering, University of South Carolina, Columbia, South Carolina, United States of America; 3 Department of Hematology and Oncology, Dr. von Hauner Children’s Hospital, Ludwig-Maximilians University, Munich, Germany; 4 Department of Pathology and Cell Biology, Herbert Irving Comprehensive Cancer Center, Columbia University Medical Center, New York, New York, United States of America; Emory University, UNITED STATES OF AMERICA

## Abstract

Despite its critical role in survival, many aspects of hematopoiesis remain unresolved. In the classical model of the hematopoietic program, quiescent hematopoietic stem cells (HSCs) sit at the top of the hematopoietic hierarchy, with the ability to self-renew and differentiate as needed. HSCs give rise to more proliferative progenitor cells, which possess multipotent potential, but have largely or completely lost self-renewal capabilities. Here, we have identified the tumor suppressor, Inhibitor of Growth 4 (ING4), as a critical regulator of multipotent progenitor (MPP) homeostasis. In the absence of ING4, we show that MPPs express a transcriptional program of hematopoietic activation, yet they remain quiescent with low levels of reactive oxygen species. Functionally, ING4-deficient MPPs are capable of robust regeneration following competitive bone marrow transplantation, resulting in substantially higher blood chimerism compared to wild-type MPPs. These data suggest ING4 deficiency promotes a poised state in MPPs, quiescent but transcriptionally primed for activation, and capable of converting the poised state into robust repopulation upon stress. Our model provides key tools for further identification and characterization of pathways that control quiescence and regeneration in MPPs.

## Background

The diverse cells of the immune system are produced through the process of hematopoiesis [1]. Stem cells capable of long-term donor engraftment are often described as long-term hematopoietic stem cells (LT-HSCs) [2,3]. LT-HSCs transition to short-term hematopoietic stem cells (ST-HSCs), which mature to multipotent progenitor cells (MPPs) [4,5]. The term “multipotent progenitor” is a general descriptor of the subset of heterogeneous HSPCs from the bone marrow (BM) that typically have lost self-renewal capability, do not contribute to serial transplantation, and are metabolically active [6–9].

Several recent studies have uncovered a significant contribution of MPPs to hematopoiesis. These studies reveal that MPPs can supply long-term hematopoietic program and function during hematopoietic recovery with minimal contribution of HSCs [7,10–15]. This body of work highlights the importance of the MPP population, but the field remains in the early stages of understanding the potential significance of these cells to hematopoiesis, particularly with regards to the mechanistic understanding of their contribution.

ING4 is a tumor suppressor protein, generally localized to the nucleus, that is associated with a high frequency of acquired, inactivating mutations and poor prognoses in diverse human cancers [16–19]. ING4 has many regulatory roles, both as a chromatin remodeling protein within the Hbo1 complex and as a direct regulator of several major signaling pathways: NF-κB, c-Myc, p53, and HIF-1α [20–25]. ING4 is highly expressed in murine hematopoietic stem and progenitor cells (HSPCs), suggesting it may have a specific role in HSPC function [26,27]. Interestingly, expression levels of ING4 in hematopoietic cells remain stable, even under stress conditions [28–31]. We have recently identified that ING4-deficient HSCs are markedly different from wild-type HSCs, and maintain a quiescent, yet poised state [32]. Here, we extend this line of inquiry by characterizing multipotent progenitor cells from the ING4^−/−^ mouse model. In the ING4^−/−^ bone marrow, MPPs are less abundant than wild-type (WT) MPPs and express an activation profile. Nevertheless, ING4^−/−^ MPPs are more quiescent than WT MPPs and do not convert the activation profile into functional activation at steady-state. Upon stress, however, ING4^−/−^ MPPs show significantly increased engraftment in sorted competitive BM transplantation compared to WT MPPs.

## Methods

### ING4^−/−^ mouse model

ING4^−/−^ mice (CD45.2) were provided by Stephen N. Jones (University of Massachusetts Medical School) and colony was maintained on site. WT C57BL/6 (CD45.2) and SJL-Ptprc^a^ Pepc^b^/BoyJ (CD45.1) mice 8–12 weeks of age served as controls. CD45.1 mice were purchased from The Jackson Laboratory. All mice were maintained at an AALAC-accredited animal facility at the University of South Carolina (USC) according to the USC Institutional Animal Care and Use Committee (IACUC) animal guidelines, and all animal experiments were performed with consent from the USC IACUC (D16-00028). This work was approved under protocol 2670-101820-071423 on 07/14/2023.

### Bone marrow collection and MPP isolation

Mice were humanely euthanized, and the long bones (femur, tibia, humerus, radius) were removed. Long bones from individual mice were crushed then filtered through a 40 μm nylon cell strainer. Cells were incubated with 1x RBC Lysis Solution (Miltenyi Biotec) to lyse red blood cells. Cells were lineage depleted via a Lineage Cell Depletion Kit (Miltenyi Biotec) using LS columns and an LS magnetic separator.

### Collection of peripheral blood

Under anesthesia, peripheral blood was collected from retroorbital venous sinus into heparinized (0.05 IU/mL; Alfa Aesar) micro-hematocrit capillary tubes (Kimble). Cells were incubated with 1x RBC Lysis Solution (Miltenyi Biotec) to lyse red blood cells.

### Antibody staining

Cells were incubated for in the dark for 30 minutes at 4°C with antibodies for MPP profiling (Lineage: B220, CD3e, CD11b, Gr-1, Ter119; Sca-1, c-Kit, CD48, CD34, CD150). Cells were then rinsed twice with phosphate-buffered saline (PBS)-bovine serum albumin (BSA). Antibodies against CD45.2 and CD45.1 were used to assess engraftment following transplantation assays. See Table 1 for complete list of antibodies and concentrations.

**Table 1:** Antibodies, clones and concentrations.

Antibodies	Clone	Color	Concentration
B220	RA3-6B2	BV510	1:200
		eFluor 450	1:100
		FITC	1:100
		PE	1:200
CD3e	145-2C11	APC-eFluor 780	1:100
		BV510	1:50
		PE	1:100
CD11b (Mac-1)	M1/70	BV510	1:200
		PE	1:100
CD16/32	93	eFluor 450	1:100
CD34	RAM34	APC	1:100
		FITC	1:30
CD45.1	A20	PE	1:100
CD45.2	104	APC	1:100
		FITC	1:100
CD48	HM48-1	BV510	1:100
		PE	1:200
CD127 (IL-7Ra)	A7R34	PE-Cyanine 7	1:200
CD150	TC15-12F12.2	APC-Cyanine 7	1:100
c-Kit (CD117)	ACK2	Alexa Fluor 700	1:200
		APC-eFluor 780	1:100
		PE-Cyanine7	1:100
		PerCP-Cyanine 5.5	1:500
Gr-1 (Ly6G/C)	RB6-8C5	BV510	1:50
		PE	1:200
		PE-Cyanine 7	1:400
Ki-67	SolA15	PerCP-Cyanine 5.5	1:2,000
Sca-1 (Ly-6A/E)	D7	APC	1:200
		BV510	1:200
Ter119	TER-119	APC-eFluor 780	1:200
		BV510	1:200
		PE	1:100

### Reactive oxygen species assay

Lineage-depleted BM cells were stained for MPP profiling, then incubated with 7.5 μM H_2_DCFDA (Invitrogen) in PBS for 30 minutes at 37°C. Cells were rinsed with PBS and analyzed by flow cytometry.

### Cell fixing and permeabilization

Lineage-depleted, stained BM cells were fixed and permeabilized with Cytofix/Cytoperm kit (Invitrogen) according to manufacturer’s instructions.

### Cell cycle characterization

Fixed and permeabilized cells were incubated in the dark overnight at 4°C with Ki-67 antibody (Invitrogen), then rinsed with PBS. Prior to analysis via flow cytometry, cells were incubated for 15 minutes at room temperature with DAPI (0.2 μL/mL, Invitrogen) and rinsed with PBS.

### Senescence assay

Lineage-depleted, stained BM cells were fixed and permeabilized with Cytofix/Cytoperm kit (Invitrogen) according to manufacturer’s instructions. Fixed and permeabilized cells were incubated with CellEvent Senescence Probe (Thermo) for 2 hours at 37ºC. Cells were rinsed with PBS. Cells were analyzed by flow cytometry.

### RNA-seq analysis

Bone marrow from 8 to 10 mice was harvested and pooled, and lineage depleted. LT-, ST-HSCs, and MPPs were sorted separately into Buffer RLT Plus (Qiagen) for both WT or Ing4^−/−^ mice. Total RNA was isolated with the RNeasy Micro column (QIAGEN). Library construction, sequencing by Illumina HiSeq, and initial bioinformatics analyses were conducted by GENEWIZ. Counts obtained were used to perform Gene Set Enrichment Analysis. Ing4^−/−^ LT-HSCs, ST-HSC, and MPPs were compared together against WT LT-HSCs, ST-HSCs, and MPPs.

### Sorted bone marrow transplant

To compare the reconstitution and maintenance capacity of ING4-deficient MPPs, sorted MPPs from CD45.2 ING4^−/−^ and WT mice were transplanted in a competitive setting. Either 100 ING4^−/−^ or 100 WT MPPs were combined with 200,000 CD45.1 unfractionated BM cells and, under anesthesia, transplanted via retroorbital injection into 8- to 12-week-old recipient mice (B6. SJL-CD45.1) that had been lethally irradiated (9.5 Gy, administered as two doses at least three hours apart). Peripheral blood and BM of recipient mice were analyzed at 12 weeks post-transplant.

### OP-puromycin staining

Lineage-depleted BM cells were stained for MPP profiling, then incubated with preheated DMEM solution containing 25 uM OP-Puromycin (Invitrogen) for 60 minutes as described previously [33]. Cells were rinsed with PBS and the Click-iT^TM^ azide-alkyne cycloaddition reaction was conducted per manufacturers protocol, then cells were analyzed by flow cytometry.

### Flow cytometry, cell sorting and analysis

Cells were analyzed on LSR II (BD), FACSAria II (BD), or FACSymphony (BD). For sorted cell assays, FACSAria II (BD) was used. FACS Diva was used in conjunction with flow cytometers for acquisition and FlowJo (version 10) was used for analysis of MPP populations.

### 5-fluorouracil treatment

Under anesthesia, 8- to 12-week-old ING4^−/−^ and WT mice received one retroorbital venous sinus injection of 5-fluorouracil (5-FU, 150 mg/kg, Sigma) or PBS. BM was harvested 15 days following 5-FU treatment, lineage depleted, stained with fluorescent antibodies, and analyzed via flow cytometry.

### Statistical analysis

Mean values ± SD are shown. Student’s t test with Welch’s correction or Mann-Whitney analysis were used for single comparisons (GraphPad Prism v.10.1.1). *= *p* < 0.05, **= *p* < 0.01, ***= *p* < 0.005, ****= *p* < 0.001. Two-way ANOVA was used for multiple comparisons for *in vivo* inhibitor assays (GraphPad Prism v.10.1.1).

## Results

### MPP differentiation is impaired in the absence of ING4

**Fig 1:**
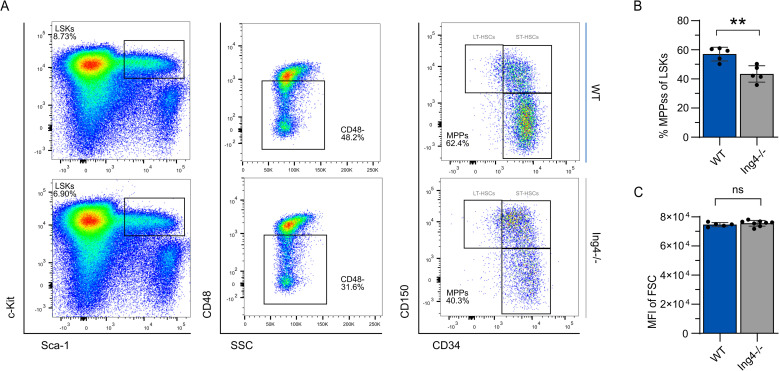
Steady-state MPP populations from BM of WT and ING4^−/−^ mice. (A) Representative gating strategy of flow cytometric analysis for MPPs. (B) MPPs from WBM isolated from individual WT and ING4^−/−^ steady state mice as a percentage of LSK cells. (*n* = 10; **=*p* < 0.01). (C) Flow cytometric analysis of cell size by FSC of MPPs isolated from individual WT and ING4^−/−^ mice. (*n* = 5–8; *=*p* < 0.05). Statistical significance was assessed using Student’s t-test with Welch’s correction.

To profile multipotent progenitor cells in the absence of ING4, we used a previously described ING4-deficient mouse model [21]. Multipotent progenitors are defined here as the Lin^−^ Sca1^+^ cKit^+^ CD48^−^ CD34^+^ CD150^−^ fraction of the whole bone marrow (Gating strategy in Fig 1A). Cell surface profiling of steady-state BM showed a significant decrease in MPPs as a percentage of LSKs observed in BM from ING4^−/−^ mice (Fig 1A and B). This may potentially result from a differentiation block at the ST-HSC stage [32]. No difference in cell size is observed between WT and ING4^−/−^ MPPs when comparing mean fluorescent indices (MFIs) of FCS profiles (Fig 1C).

### ING4-deficient MPPs show characteristics of quiescence

Quiescence is a state in which the cell has the ability to divide, but largely remains in a resting state under steady-state conditions [34]. Cells can enter the quiescent, dormant G_0_ phase from G_1_, thereby minimizing susceptibility to mutations in the DNA acquired through cell division and maintain long-term preservation of hematopoiesis [35,36]. MPPs are typically more metabolically active, and spend more time in G_1_ than HSCs [7].

**Fig 2:**
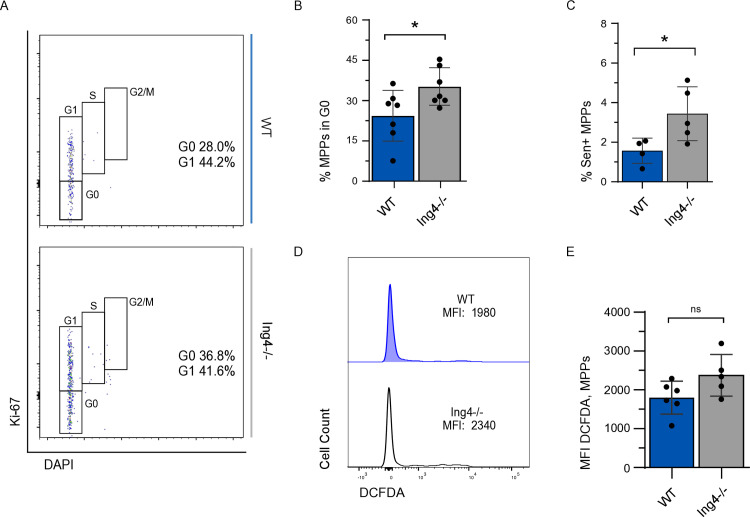
ING4-deficient MPPs have increased quiescence and low ROS levels. (A) Representative flow cytometric analysis of MPPs using Ki-67 and DAPI for cell cycle profile. (B) MPPs in G_0_ isolated from BM of individual WT and ING4^−/−^ steady-state mice as a percentage of the MPP population. (*n* = 4–7; *= *p* < 0.05). (C) Senescence-positive MPPs isolated individual WT and ING4^−/−^ steady-state mice as a percentage of all MPPs. (*n* = 4–5; *= *p* < 0.05). (D) Representative flow cytometric analysis of staining with DCFDA for ROS in MPPs from BM of WT and ING4^−/−^ mice. (E) MFI of DCFDA in MPPs isolated from WT and ING4^−/−^ steady-state mice. (*n* = 5–6; ns = *p* > 0.05). Statistical significance was assessed using Student’s t-test with Welch’s correction.

To examine cell cycle status in the absence of ING4, 4’-6-diamidino-2-phenylindole (DAPI) was used to visualize DNA content and Ki-67 showed proliferative status. When compared to WT, cell cycle status of ING4^−/−^ MPPs showed an increased proportion of cells in G_0_ (Fig 2A and B).

Senescence is defined as irreversible, stable cell cycle arrest and happens in response to intrinsic or extrinsic stressors. Because Ki-67 only differentiates between cycling versus non-cycling cells, it cannot discriminate between quiescent and senescent cells, as cells in neither state will incorporate Ki-67. Analysis of senescence-associated β-galactosidase (SA-β-gal) staining of WT and ING4^−/−^ MPPs revealed a small but significant increase in percentage of senescence-positive MPPs (1.6% and 3.4%, respectively) (Fig 2C). The relatively small proportions of senescent-positive cells found here likely do not account for the difference in proportions of MPPs observed to be in the G_0_ phase of the cell cycle, suggesting that MPPs from ING4^−/−^ mice are more quiescent than WT.

As low intracellular reactive oxygen species (ROS) content is associated with quiescence [37,38], MPPs were assayed to determine if ROS levels differed between WT and ING4^−/−^ MPPs. Lineage-depleted BM cells were treated with 2’7’-deichlorofluorescein diacetate (H_2_DCFDA) to examine ROS levels in WT and ING4^−/−^ MPPs. ROS levels, as indicated by MFI, showed no significant difference between WT and ING4^−/−^ MPPs (D and E).

To investigate if loss of ING4 impacts apoptosis in MPPs and may account for the reduction in MPPs observed in the absence of ING4, we determined the frequency of annexin V^+^ MPPs undergoing apoptosis in WT and ING4-deficient populations. The percentage of MPPs undergoing apoptosis was similar between WT and ING4^−/−^ mice (S1 Fig).

### ING4^−/−^ MPPs simultaneously express genes associated with activation and quiescence

**Fig 3:**
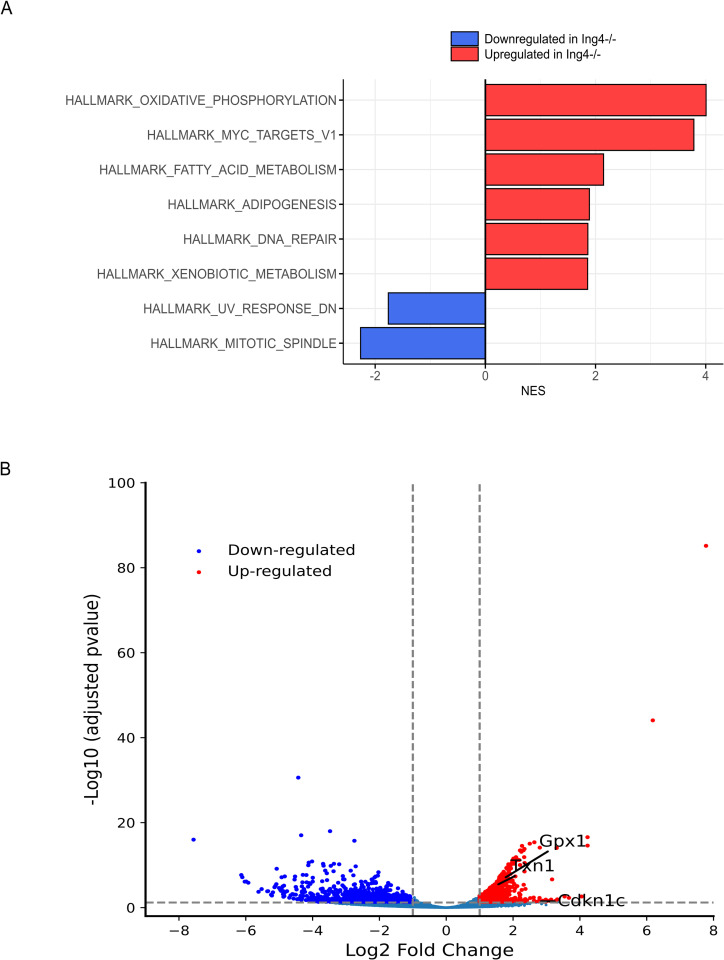
Loss of ING4 promotes expression of a hematopoietic activation profile in MPPs. (A) Normalized Enrichment Scores (NES) from a Hallmark gene set analysis comparing populations (WT LT-HSCs, ST-HSCs, and MPPs pooled), and (Null LT-HSCs, ST-HSCs, and MPPs pooled). A total of 8 enrichment pathways (FDR-adjusted *p*-value < 0.05) were identified. Bar colors indicate statistical significance. (B) Differentially expressed genes in pooled WT HSPCs compared to pooled ING4^−/−^ MPPs, with a statistically significant threshold of *p* < 0.05, and a Log_2_Fold change = 1. Upregulated genes are represented in red, downregulated in blue.

To elucidate the molecular consequences of ING4 loss in HSPC regulation, we conducted a genome-wide expression analysis using bulk RNA-sequencing (RNA-seq) of purified ING4^−/−^ LT-HSCs (LSK CD48^−^CD34^−^CD150^+^), ST-HSCs (LSK CD48^−^CD34^+^ CD150^+^) and MPPs. We then compared the profiles of these populations, pooled, against pooled wild-type LT-HSC, ST-HSC, and MPP gene expression. This analysis provided a way to identify stem cell-like gene signatures in the MPP populations. We revealed 1,635 differentially expressed genes in the combined HSPC populations (936 upregulated and 699 downregulated, *p* < 0.05). Surprisingly, Gene Set Enrichment Analysis (GSEA) of this data set using the Hallmark gene set showed common upregulated genes in ING4^−/−^ HSPCs were associated with oxidative phosphorylation (OXPHOS), ribosomal biogenesis (RiBi), and c-Myc target gene expression (Fig 3A). Downregulated genes were associated with mitotic spindle formation, and UV response (Fig 3A). All dysregulated genes are represented in a volcano plot shown in Fig 3B. A set of genes that are associated with quiescence and cell cycle regulation were also upregulated, including the cell-cycle regulator *p57*, *txn1* and *gpx1* (Fig 3B).

### ING4^−/−^ MPPs have normal levels of translation and OXPHOS utilization

**Fig 4:**
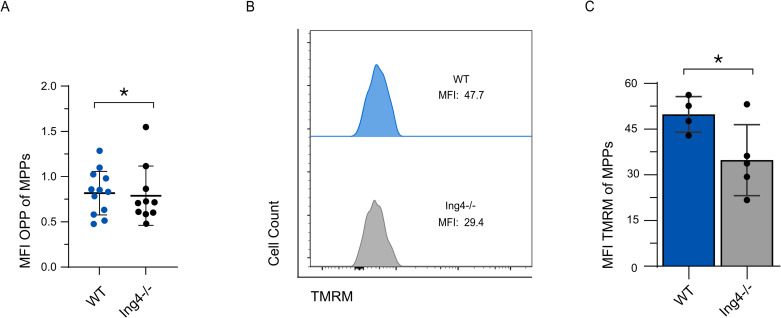
ING4-deficient MPPs have normal translation rates and reduced mitochondrial potential. (A) MFI of OPP-treated MPPs isolated from individual WT and ING4^−/−^ steady-state mice. (*n* = 10–23; *= *p* < 0.05). (B) Representative flow cytometric analysis of staining for TMRM in MPP populations in WT and ING4^−/−^ WBM at steady-state. (C) MFI of TMRM-treated MPPs isolated from individual WT and ING4^−/−^ steady-state mice. (*n* = 5; *= *p* < 0.05). Statistical significance was assessed using Student’s t-test with Welch’s correction for (A) and Mann-Whitney analysis (C).

Based on the RNA-sequencing data, we next sought to determine if ING4^−/−^ MPPs are more metabolically active, as their transcriptional profile would suggest. Hematopoietic activation is associated with increased translation [39]. To examine if an increase in translation rate accompanies the increase in RiBi-associated genes upregulated in our RNA-seq data set, we used O-propargyl-puromycin (OP-Puro) incorporation. To this end, lineage depleted BM cells were treated with OP-Puro for 1 hour, then analyzed for levels of OP-Puro incorporation. Surprisingly, these assays show no increase in the translation rates of ING4^−/−^ MPPs (Fig 4A).

To further address ING4^−/−^ MPP activation, we examined the OXPHOS pathway, as components of this pathway also show transcriptional activation. As HSPCs are activated, there is a characteristic shift from glycolysis to oxidative phosphorylation. To profile metabolic activation, we examined tetramethylrhodamine methyl ester (TMRM) levels, which indicate mitochondrial membrane potential. As activated cells transition from glycolysis to oxidative phosphorylation to generate ATP, an electrical potential gradient forms across the mitochondrial membrane and is observable in TMRM staining. We observed a significant decrease in MFI in ING4^−/−^ MPPs as compared to WT counterparts, suggesting that ING4^−/−^ MPPs are not using OXPHOS to generate higher levels of ATP than WT MPPs (Fig 4B and C). The delocalization of TMRM in the mitochondria indicated by the decreased MFI in the ING4^−/−^ MPPs suggests they may rely more heavily on glycolysis to generate ATP than WT MPPs.

Together, these signatures suggest that ING4 deficiency may promote a transcriptionally poised state in MPPs, whereby ING4 loss upregulates genes associated with activation while also inducing quiescence-associated genes to maintain MPPs in a resting state.

### ING4^−/−^ MPPs contribute to multilineage engraftment at higher levels than WT MPPs

**Fig 5:**
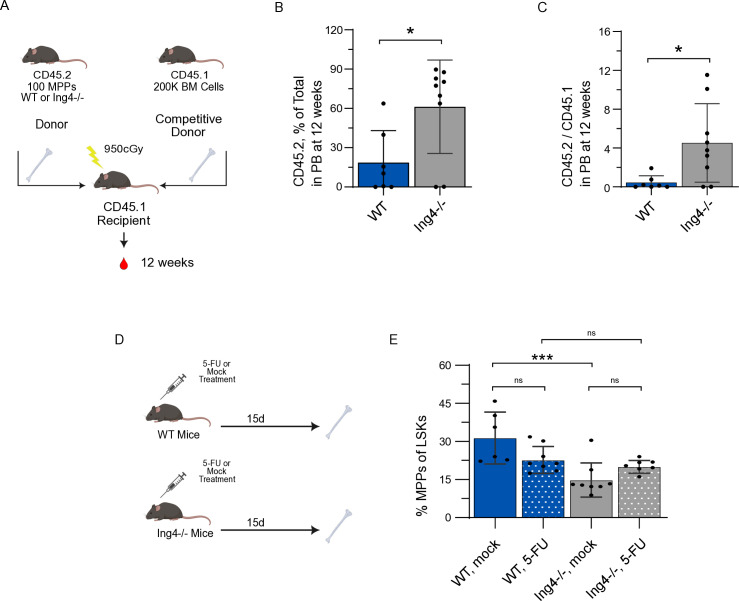
In a sorted, competitive bone marrow transplantation assay, ING4^−/−^ MPPs contribute to chimerism at higher levels than WT MPPs and respond more robustly to 5-fluorouracil exposure. (A) Schematic overview of the sorted, competitive transplantation assay using BM from WT and ING4^−/−^ mice. (B) CD45.2 chimerism in individual MPP-recipient mice from peripheral blood collected 12 weeks following sorted, competitive transplantation from WT or ING4^−/−^ mice. (*n* = 7-9; *= *p* < 0.05). (C) Ratio of CD45.2/CD45.1 cells in individual MPP-recipient mice from peripheral blood collected 12 weeks following sorted, competitive transplantation from WT or ING4^−/−^ mice. (*n* = 7–9; *= *p* < 0.05). (D) Schematic overview of the 5-FU assay in WT and ING4^−/−^ mice. (E) MPPs isolated from individual 5-FU or mock treated WT and ING4^−/−^ mice, as a percentage of the LSK population at 15 days post-treatment. (*n* = 6–8; ns = *p* > 0.05; ***= *p* < 0.005). Statistical significance was assessed using Mann-Whitney analysis.

To compare the engrafting capacities of ING4^−/−^ and WT MPPs, we sorted MPPs from CD45.2^+^ ING4^−/−^ and WT donor mice. Separately, pools of either 100 ING4^−/−^ MPPs or 100 WT MPPs were transplanted, along with 200,000 CD45.1+ competitor marrow cells into CD45.1^+^ recipient mice (Fig 5A). Peripheral blood was analyzed at 12 weeks post-transplant and revealed a significant increase of donor contribution from the ING4^−/−^ MPPs than WT (61.3% and 18.6%, respectively) (Fig 5B). Furthermore, ING4^−/−^ MPPs were shown to reconstitute the myeloid and T-cell populations at a significantly higher rate than WT MPPs (S2 Fig). Transplanted ING4^−/−^ MPPs also made a greater contribution to B-Cell population than WT MPPs, though this difference was not statistically significant.

We also examined the response of ING4^−/−^ MPPs to hemopoietic stress following treatment with the myeloablative chemotoxin 5-fluorouracil (5-FU). Mice were treated with a single dose of 5-FU or mock treatment and analyzed at 15 day post exposure (15 d) (Fig 5D). We found that at 15 d post-treatment, WT mice who received 5-FU had a slight decrease in MPPs as a percentage of LSKs, while ING4^−/−^ mice had an increase in MPPs as a fraction of LSKs, suggesting an expansion of ING4^−/−^ MPPs in response to 5-FU exposure (Fig 5E). Taken together, these data suggest that loss of ING4^−/−^ results in a more robust response to hematopoietic stress in ING4^−/−^ MPPs than WT MPPs.

## Conclusions

The mechanisms that regulate maintenance and proliferation of hematopoietic stem and progenitor cells have yet to be clearly defined. Here, we have uncovered a role for ING4 in regulation of the potency of MPPs. No role for ING4 has previously been identified in MPPs. As MPPs make up a higher proportion of the BM than HSCs, and we have identified ING4^−/−^ MPPs as showing enhanced engraftment capabilities, understanding the differences in ING4^−/−^ MPPs as compared to WT counterparts may provide important avenues for clinically relevant advancements in treatment. Here we characterized some key aspects of ING4^−/−^ MPPs. Similar to our work in HSCs [32], we found that loss of ING4 results in a poised state, where MPPs express a program of transcriptional activation, but are quiescent with low ROS levels and reduced mitochondrial membrane potential, all hallmarks of a quiescent population [32].

Loss of ING4 results in a decreased proportion of MPPs from the LSK compartment as compared to WT MPPs, providing evidence of skewed hematopoiesis. MPPs from ING4^−/−^ mice are more quiescent than WT counterparts, with a much larger increase in non-cycling cells than can be accounted for by the SA-β-gal^+^ senescent population. Previously studies have shown that ING4 is involved in acetylation of H4 on Lys5, Lys8 and Lys12 in cooperation with the HBO1 complex, impacting cell cycle progression [22,40]. Furthermore, ING4 has been shown to induce p300-mediated acetylation of p53, promoting cells’ transition from G_1_ to S phase [18,40]. Although these studies were not done in hematopoietic cells, they show that ING4 may impact cell cycle regulation in part through association with the Hbo1 complex. For ING4^-/-^ MPPs, no change in p53 or p21 expression was observed and apoptosis rates are similar to WT, but we did note increased expression of CDKN1c (p57), suggesting the mechanism of cell cycle regulation in hematopoietic cells may be different than other tissue types. Understanding how ING4 regulates cell cycle and if this regulation is direct will be a major focus of future studies.

Transcriptomic analysis of ING4-deficient HSPCs reveals that these cells resemble activated progenitors rather than quiescent cells [41]. Notably, genes related to oxidative phosphorylation, ribosomal biogenesis, and c-Myc targets are significantly overexpressed, aligning their profiles with those of activated progenitors. When considered with the quiescent state of ING4-deficient MPPs, these data suggest that despite expression of a transcriptional activation program, the quiescent state of these cells may inhibit the transition to full activation. Future work will focus on uncoupling the transcriptional activation program and the quiescence phenotype observed in ING4^-/-^ MPPs to understand how the unexpected transcriptional profile arises. This poised state has a striking effect on MPPs under stress. After sorted transplantation, ING4-deficient MPPs from ING4^−/−^ donors engraft at much higher levels than WT MPPs and are able to support multilineage regeneration of the hematopoietic system. ING4^−/−^ MPPs also show a more robust response to 5-FU treatment at 15 d post-treatment.

Few models of enhanced MPP activity have been previously identified. Introduction of NUP98-HOXA10hd into MPPs resulted in long-term reconstitution up to 44-weeks post-transplant, similar to our observations with ING4^−/−^ MPPs. Furthermore, ectopic Sox17 expression in adult HSCs and MPPs also confers increased self-renewal potential, such that these HSPCs are more reminiscent of fetal HSPCs [42]. Sox17 expression in adult HSPCs allowed for long-term multilineage reconstitution of the hematopoietic system following transplantation, though skewed toward myelopoiesis and erythropoiesis, which, again, bears resemblance to fetal hematopoiesis patterns [42]. Finally, ectopic expression of miR-125a in murine and human multipotent progenitors also resulted in enhanced self-renewal and long-term multilineage engraftment following serial transplantation [43–46]. Although the pathways altered in these different models are largely varied amongst each other and our model, taken together, these data provide strong evidence of the potential for MPPs to have an impact on enhancing hematopoietic recovery.

Recent work in characterization of MPP4 (LSK CD135^+^ CD48^+^ CD150^−^) and 5 (LSK CD135^−^ CD48^−^ CD150^−^) subpopulations suggest MPPs can retain an increased capacity for quiescence and differentiation, and further highlight the contributions of MPP subpopulations to the maintenance of the hematopoietic system under steady-state conditions [7,41]. Together, these data reveal distinct subpopulations of MPPs as components of the broader heterogeneous MPP pool that possess the capacity to regenerate at substantial levels.

Understanding how loss of ING4 may enhance the function of the specific MPP subpopulations may have clinical significance and warrant continued study. In future work, we will use the ING4^-/-^ model to dissect and identify the ING4 specific mechanisms that contribute to enhanced regeneration, with the ultimate goal of identifying targetable pathways that can be modulated to improve hematopoietic function in transplantation. Additionally, we may also distinguish a pool of MPPs could be used as a supplemental support for improved recovery from BMT, as a mechanism for rapid transient, replenishment during the engraftment period.

### Limitations of study

MPPs are described by the presence of a wide variety of cell surface markers that can be profiled by flow cytometry. While we have defined MPPs as Lin^−^ Sca-1^+^ c-Kit^+^ CD150^−^ CD34^+^, we have not used CD135, CD244 or CD229, which are additional commonly used cell surface markers in isolating subpopulations of HSPCs. This, coupled with deeper analysis of RNA from ING4^−/−^ MPPs, would likely provide insight into molecular pathways that may confer this cell population with its unique characteristics.

Additionally, the transplantation assay was only performed once. Although this assay was robust with 7–9 mice per treatment, repetition of this assay would strengthen the findings. Taken together with the finding that 5-FU exposure leads to ING4^−/−^ MPP expansion, these data suggest that ING4 confers an enhanced capacity to respond to hematopoietic stress. Future studies will examine longer term responses for both 5-FU and transplantation.

## Supporting information

S1 Fig MPPs from ING4^−/−^ and WT mice undergo similar rates of apoptosis. Percentage of Annexin V^+^ MPPs of individual WT and ING4^−/−^ steady-state mice. (*n* = 3–4; ns = *p* > 0.05). Data in S1 reflect mean values ± SD. Statistical significance was assessed using Mann-Whitney analysis.
(TIF)

S2 Fig Control staining for senescence, reactive oxygen species, and mitochondrial membrane potential in control samples. (A) Representative flow cytometric analysis of unstained (blue) and all cells (red) stained with beta-galactosidase for senescence. (B) Representative flow cytometric analysis of unstained (blue) and all cells (red) stained with DCFDA. (C) Representative flow cytometric analysis of unstained (blue), all cells treated with CCCP and stained with TMRM (purple) and all cells stained with TMRM (red) for mitochondrial potential. (*n* = 4–7).
(TIF)

S3 Fig Bone Marrow chimerism of individual lineages shows mild lineage bias towards myeloid and T cells. (A) CD45.2 chimerism of myeloid cells in individual MPP-recipient mice from peripheral blood collected 12 weeks following sorted, competitive BM transplant from WT or ING4^−/−^ mice. (*n* = 7–9; *= *p* < 0.05). (B) CD45.2 chimerism of B-cells in individual MPP-recipient mice from peripheral blood collected 12 weeks following sorted, competitive BM transplant from WT or ING4^−/−^ mice. (*n* = 7–9; ns = *p* > 0.05). (C) CD45.2 chimerism of T-cells in individual MPP-recipient mice from peripheral blood collected 12 weeks following sorted, competitive BM transplant from WT or ING4^−/−^ mice. (*n* = 7–9; *=*p* < 0.05). Data reflect mean values ± SD. Statistical significance was assessed using Mann-Whitney analysis.
(TIF)
