# Supplementary figures and images for: Loss of ING4 enhances hematopoietic regeneration in multipotent progenitor cells
Source: PLoS One. 2025 Feb 14;20(2):e0316256. doi: 10.1371/journal.pone.0316256 (PMC11828401; doi:10.1371/journal.pone.0316256)

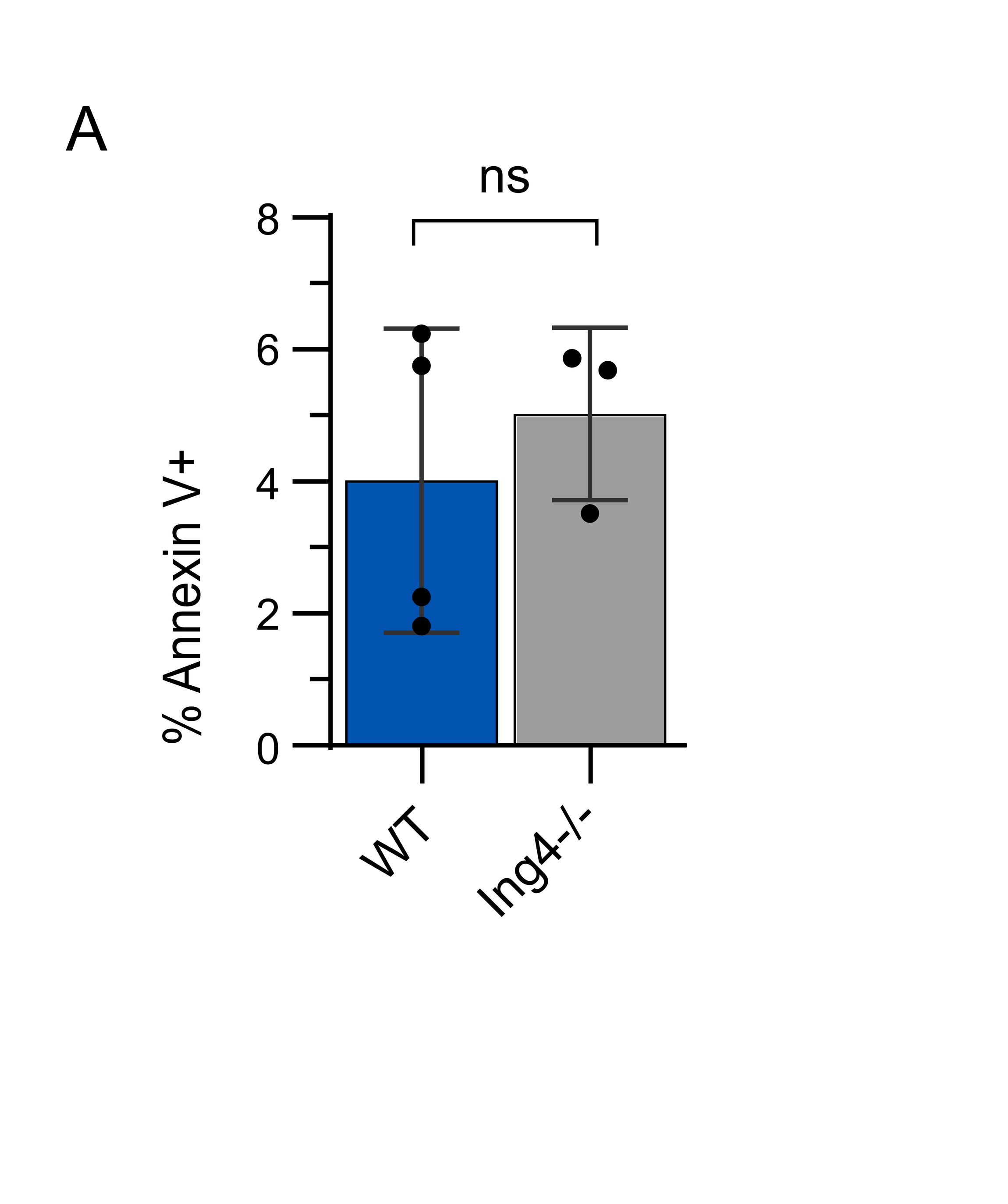

Supplement: S1 Fig — Percentage of Annexin V+ MPPs of individual WT and ING4−/− steady-state mice. (n = 3–4; ns = p > 0.05). Data in S1 reflect mean values ± SD. Statistical significance was assessed using Mann-Whitney analysis. (TIF) [file pone.0316256.s001.tif]

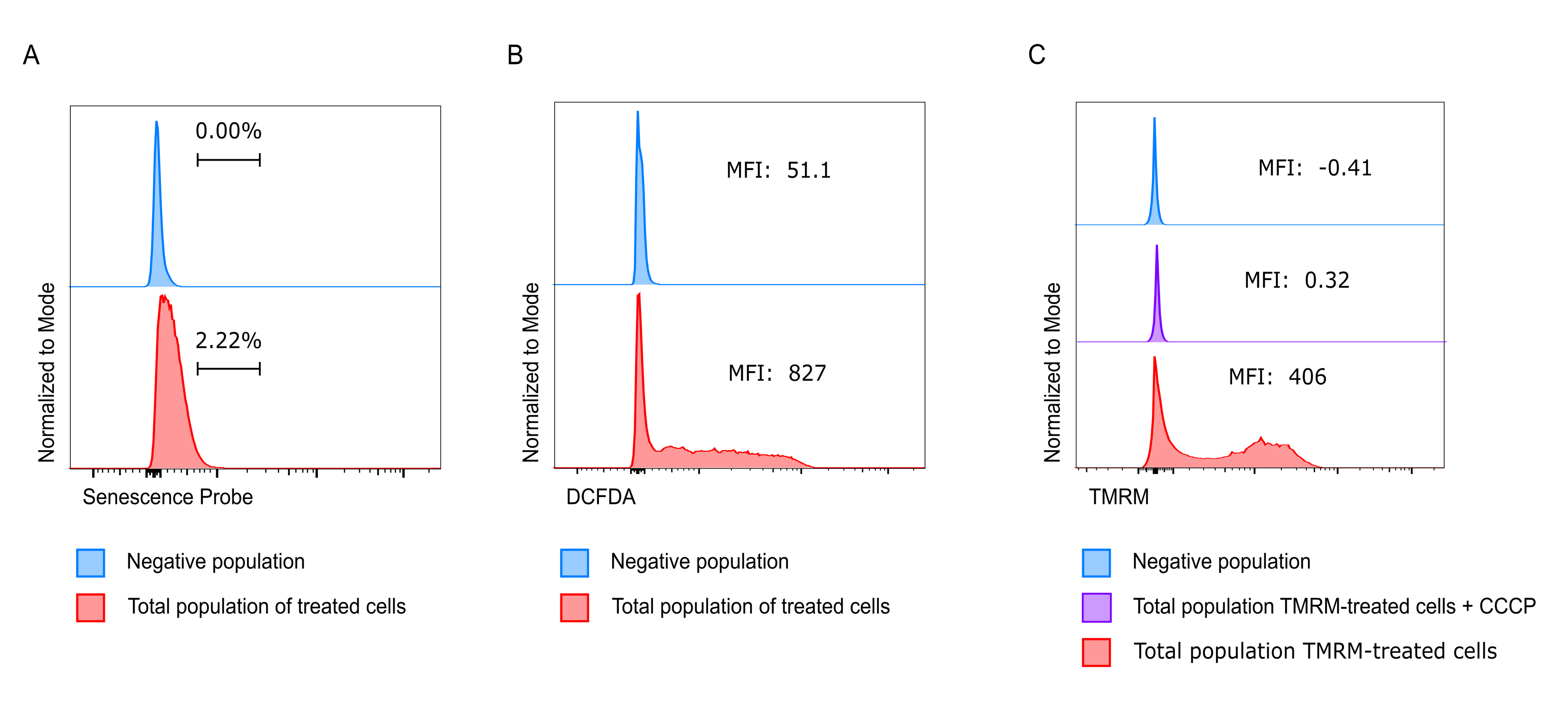

Supplement: S2 Fig — (A) Representative flow cytometric analysis of unstained (blue) and all cells (red) stained with beta-galactosidase for senescence. (B) Representative flow cytometric analysis of unstained (blue) and all cells (red) stained with DCFDA. (C) Representative flow cytometric analysis of unstained (blue), all cells treated with CCCP and stained with TMRM (purple) and all cells stained with TMRM (red) for mitochondrial potential. (n = 4–7). (TIF) [file pone.0316256.s002.tif]

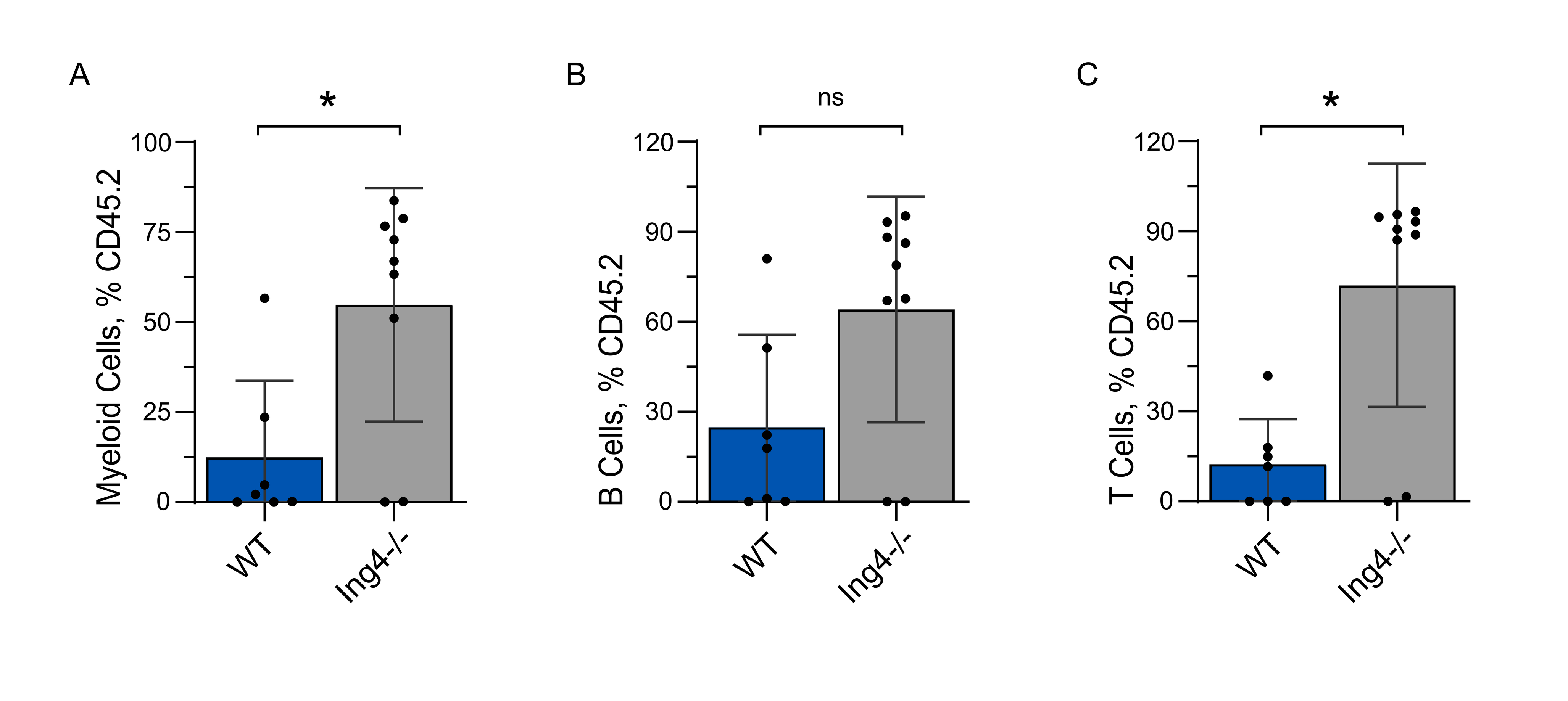

Supplement: S3 Fig — (A) CD45.2 chimerism of myeloid cells in individual MPP-recipient mice from peripheral blood collected 12 weeks following sorted, competitive BM transplant from WT or ING4−/− mice. (n = 7–9; *= p < 0.05). (B) CD45.2 chimerism of B-cells in individual MPP-recipient mice from peripheral blood collected 12 weeks following sorted, competitive BM transplant from WT or ING4−/− mice. (n = 7–9; ns = p > 0.05). (C) CD45.2 chimerism of T-cells in individual MPP-recipient mice from peripheral blood collected 12 weeks following sorted, competitive BM transplant from WT or ING4−/− mice. (n = 7–9; *=p < 0.05). Data reflect mean values ± SD. Statistical significance was assessed using Mann-Whitney analysis. (TIF) [file pone.0316256.s003.tif]
